# NCAPD3-mediated ferroptosis of 2,3,5,4’-Tetrahydroxystilbene-2-O-β-D-glucoside inhibits proliferation in T47D cells

**DOI:** 10.3389/fphar.2024.1531220

**Published:** 2025-01-29

**Authors:** Jianfen Shen, Shuo Zhang, Yan Song, Leiming Yang, Qi Huang, Pengyu Wang, Youzhi Zhang

**Affiliations:** ^1^ Department of Central Laboratory, The Affiliated Hospital of Jiaxing University, Jiaxing, Zhejiang, China; ^2^ School of Pharmacy, Hubei Engineering Research Center of Traditional Chinese Medicine of South Hubei Province, Xianning Medical College, Hubei University of Science and Technology, Xianning, China; ^3^ Hubei Key Laboratory of Diabetes and Angiopathy, Hubei University of Science and Technology, Xianning, China

**Keywords:** THSG, NCAPD3, breast cancer, ferroptosis, full-length transcriptome sequencing, T47D cells

## Abstract

**Objective:**

Non-SMC condensin II complex subunit D3 (NCAPD3) has recently been demonstrated as a crucial oncogenic factor, nevertheless, the biological role of NCAPD3 in the pathogenesis of breast cancer has not been elucidated. Evidence suggests that targeting ferroptosis can inhibit the progression of breast cancer. Moreover, 2,3,5,4’-Tetrahydroxystilbene-2-O-β-D-glucoside (THSG) could modulate MCF-7 cell proliferation in our previous study. Therefore, we aimed to investigate the potential mechanism by which NCAPD3 mediates ferroptosis in THSG inhibition of T47D cell proliferation by full-length transcriptome sequencing.

**Methods:**

Alternative splicing analysis was performed based on full-length transcriptome sequencing and the overlapping genes in differentially expressed transcripts (DETs) and differential alternative splicing (diAS) were obtained. Further, RT-PCR was used to validate the type of alternative splicing. And the hub genes (transcripts) were selected using the bioinformatics analysis, quantitative polymerase chain reaction (qPCR) and Western blotting (WB). Moreover, cell cycle and ferroptosis were assessed using flow cytometry analysis and WB respectively. Mechanically, cell viability and clone formation was detected using Biochemical kit. And siRNA of *Ncapd3* was transfected into T47D cells to detect the expression levels of ferroptosis-related proteins (WB) and cell viability (MTT).

**Results:**

40 overlapping transcripts of DETs and diAS were obtained consistent with the analysis of full-length transcriptome sequencing, and *Ncapd3* (*Ncapd3-203*) is key gene (transcript), which was also highly expressed in breast cancer and THSG could inhibit the mRNA and protein expression. Moreover, THSG could induce cell cycle arrest in G2/M stage and reduce ferroptosis-related protein expression (xCT and GPx4). Mechanically, we found that THSG inhibits the cell proliferation and clone formation in T47D cells, and *Ncapd3* inhibition could inhibit (xCT and GPx4) proteins expression, which regulated THSG-suppressing effect in T47D cells.

**Conclusion:**

THSG could inhibit the proliferation in T47D cells by NCAPD3 -dependent ferroptosis, which provided novel insights into targeted strategy for breast cancer.

## 1 Introduction

Global cancer statistics in 2024 released breast cancer ranks first in incidence among women worldwide and second in mortality. It has become the leading malignant tumor that severely threatens women’s physical and mental health (Siegel RL et al., 2024; Yang Z et al., 2024). Studies have shown that 60%–70% of breast cancers are estrogen receptor-positive (ER+) and progesterone receptor-positive (PR+) hormone-dependent malignant tumors (Siegel RL et al., 2023; Trabert B et al., 2020). The prevalence of patients with ER + breast cancer who relapse and metastasise after 5 years remains at 5%–30% (Nanda A et al., 2021). MCF-7 and T47D, as ER + breast cancer cell line indicators, significantly contribute to the advancement of breast cancer. Chemotherapy, radiation, and endocrine therapy possess specific benefits in eradicating tumour cells and suppressing tumour proliferation, nonetheless, they frequently entail significant adverse effects and the development of drug resistance. Consequently, identifying novel molecular targets associated with the progression of ER + breast cancer is crucial for the targeted therapy of patients.

2,3,5,4’-Tetrahydroxystilbene-2-O-b-D-glucoside (THSG) is the main active ingredient in the traditional Chinese medicine *Polygonum multiflorum* (Wu J et al., 2017). In our previous studies, the combination of THSG and doxorubicin exerted a synergistic effect on MCF-7 cells through the PI3K/Akt pathway and induced apoptosis (Shen J et al., 2018). We further studied and found that THSG regulates the alternative splicing of CHEK2 and CCND1 by inducing G0/G1 cell cycle arrest, thereby inhibiting MCF-7 cell proliferation (Shen H et al., 2024). Evidence demonstrates that T47D cell lines exhibit greater sensitivity to progesterone than MCF-7 cell lines, that supports further investigation into T47D cell lines (Yu S et al., 2017). At the moment, there is not any evidence about the inhibitory action of THSG on T47D, consequently THSG may potentially emerge as a novel therapeutic agent that blocks the proliferation of T47D cells.

Non-SMC condensin II complex subunit D3 (NCAPD3) is located at 11q25, which contains 37 exon sequences, 1498 amino acids, 4 HEAT repeat domains and a coiled-coil domain (Yeong FM et al., 2003). Dysfunction of NCAPD3 can lead to disruptions in chromosome condensation and errors in segregation. In recent years, NCAPD3 has been intricately linked to cancer development and progression, particularly in colorectal cancer (Jing et al., 2022a), prostate cancer (Jing et al., 2022b), gastric cancer (Zhang SY et al., 2024), and non-small cell lung cancer (Yang F et al., 2024). However, its mechanisms in breast cancer remains unclear. Further investigation may provide a theoretical foundation for breast cancer prevention and treatment.

Ferroptosis was novel way to induce cell death that is iron-dependent and morphologically and biochemically different from autophagy, apoptosis, and necrosis. It is primarily characterized by iron accumulation and lipid peroxidation (Dixon SJ et al., 2012). Ferroptosis is regulated by various factors, among which Glutathione Peroxidase 4 (GPx4) plays a crucial role in modulating lipid peroxidation. GPX4 is markedly overexpressed in breast cancer, where its primary function is to neutralize reactive oxygen species (ROS) by converting glutathione (GSH) into its oxidized form, glutathione disulfide (GSSG) (Shi Z et al., 2021; Lin HY et al., 2021; Wang D et al., 2021). Moreover, Solute Carrier Family 7 Member 11 (SLC7A11, xCT) is a crucial component in the production of glutathione (GSH) and is located upstream of ferroptosis. Studies indicate that the inhibition of SLC7A11 may prevent the spread of breast cancer cells (Liu J et al., 2020). Consequently, we conclude that the stimulation of ferroptosis may serve as a mechanism that suppresses breast cancer cells and enhances the effectiveness of anti-tumor agents and radiotherapy.

Therefore, this article will be studied in three sections. First, *Ncapd3* (*Ncapd3-203*) is key alternative splicing gene, which was also highly expressed in breast cancer. Secondly, THSG could inhibited the NCAPD3 protein levels and activated ferroptosis and cell cycle arrest in T47D cells. Collectively, targeted inhibition of *Ncapd3* could trigger ferroptosis, which regulates THSG-suppressing effect in T47D cells.

## 2 Materials and methods

### 2.1 Preparation of THSG and cell culture

THSG was obtained from Chengdu Herbpurify CO., LTD (Cat#E−022-160,001, Chengdu, China) and dissolved in Roswell Park Memorial Institute 1640 (RPMI 1640, Cat#12633020, Gibco, United States) with 2% fetal bovine serum (FBS) (Cat#FSD500, Excell, Jiangsu, China) at a concentration of 10 mmol/L stock solution. The concentrations used in this study were 0, 100, 200, 300, 400, and 500 μmol/L, freshly diluted in RPMI 1640 medium before use. The concentration of 0 μmol/L THSG was set as control.

### 2.2 Cell culture and transfection

T47D cells (Cat#BFN60805678, ATCC) cultured in RPMI 1640 supplemented with 10% FBS and 1% penicillin as well as streptomycin (PS, Cat#G4003, Servicebio, Wuhan, China). The cells were cultured at 37 °C with 5% CO_2_ in humidified conditions. Transfection was performed with Lipofectamine 2000 (Cat#11668030, Invitrogen, Carlsbad, CA, United States) according to the manufacturer’s instructions. For RNA interference, cells were transfected with appropriate siRNAs using Lipofectamine RNAiMAX (Cat#13778075, Invitrogen, Carlsbad, CA, United States) and harvested 48 h later for analyses. With subsequent examinations, T47D cells was transfected with scramble siRNA (Cat#A06002, Shanghai GenePharma Co., Ltd. Shanghai, China) and *Ncapd3* siRNA. (*Ncapd3* siRNA sense, 5-GUG​CUG​CCU​UUC​ACU​UUA​ATT-3, and its antisense siRNA was 5-UUA​AAG​UGA​AAG​GCA​GCA​CTT-3, Cat#A03001, Shanghai GenePharma Co., Ltd. Shanghai, China). Then, T47D cells line was collected and the mRNA expression of *Ncapd3* were measured using quantitative polymerase chain reaction (qPCR) in 48 h later.

### 2.3 Cell viability assay

The proliferation and cytotoxicity of T47D cells was assessed using the methyl thiazolyl tetrazolium (MTT) assay (Cat#C0009S, Beyotime Biotechnology, Shanghai, China). T47D cells were seeded at a cell density of 5 × 10^3^ cells/well in 96-well plates and cultured with different concentrations (0, 100, 200, 300, 400, and 500 μmol/L) of THSG were added for 24, 48, and 72 h. The cells were cultured for 4 h after the addition of MTT solution (10 μL per well). Subsequently, 100 μL of dimethyl sulfoxide (DMSO, Cat#ST038, Beyotime Biotechnology, Shanghai, China) per well was added, and the mixture was incubated for 10 min while the cells were shaken. Relative absorbance was determined at 570 nm by subtracting the absorbance at 630 nm. Inhibition rate (%) = 100% × (control cell OD -dosing cell OD)/(control cell OD-blank OD).

### 2.4 Colony formation assay

T47D cells (1 × 10^2^) with 100–200 μmol/L THSG or Phosphate Buffered Saline (PBS) (Cat#G4202, Servicebio, Wuhan, China) were inoculated and incubated in 6-well plates for 24 h. The cells were subsequently cultivated in mediums lacking THSG for 2 weeks. All of the dishes were subsequently rinsed multiple times with ddH2O to eliminate unattached crystal violet prior to enumerating the colonies employing ImageJ 1.53a software.

### 2.5 Cell cycle detection

Cell cycle was evaluated using a related Kit (Cat#C1052, Beyotime Biotechnology, Shanghai, China) as follows: T47D cells were treated to various doses of THSG (0, 200, and 250 μmol/L) for a duration of 24 h. Subsequently, the cells were treated with a concentration of 200 μmol/L THSG for 24 and 48 h. Afterwards, the cells were subjected to centrifugation and re-suspended in PBS. The cells were stained with propidium iodide (PI) following the instructions provided by the manufacturer. The data were acquired using flow cytometry equipment (LSRFortessa, United States) and processed using FlowJo-V10 software (Tree Star Inc.).

### 2.6 Full-length transcriptome sequencing was constructed for diAS, DEGs and DETs in T47D cells

Third-Generation Sequencing (TGS) was commercialized by Oxford Nanopore Technologies (ONT) Minions. T47D cells including control group (T47DC) and THSG-treated group (T47DT) pretreated with THSG (0 or 200 μmol/L) for 24 h, with three replicates per group. Total cellular RNA was extracted and mailed to Beijing Biomarker Technology Co., Ltd. for sequencing analysis. The raw data format used by Oxford Nanopore Technologies (ONT) is the second-generation fast5 format, which stores the original sequencing signals generated during the sequencing process. The data in fast5 format was transformed to fastq format using the Guppy software included in the MinKNOW 2.2 package. The consistent sequences of each sample were aligned with the reference genome by minimap2. Perform redundant-remove for alignment result, filter out sequences with identity lower than 0.9 and coverage lower than 0.85, consistent sequences of each sample can be used for alternative splicing analysis after redundant-remove. finally 44,416 redundant-removed transcript sequences can be obtained. It's recommended to use IGV (Integrative Genomics Viewer) to open alignment result file between transcriptome sequencing Reads and reference genome sequence (usually in BAM format), species reference genome sequence and the annotation file for visual browsing.

The data were downloaded from the BMK Cloud (https://international.biocloud.net). To detect valid alternative splicing events, those with a *P* < 0.05 and |△PSI| >10% were categorized as differential alternative splicing (diAS) events. For exons in alternative splicing, percent-spliced-in (PSI) was calculated as PSI = Splice in/(Splice in+ Splice out). “Splice-in” and “splice-out” represent the number of reads that corroborate the occurrence of splice-in and splice-out, respectively, in the RNA-seq data. All pairwise comparisons were assessed using the DESeq2 R package (1.6.3) to DEGs and DETs. In the DESeq2 analysis, differentially regulated genes were defined as those with a two-fold change, with an adjusted *P* < 0.05. Following that, the data that had been chosen were used for visualisation by being loaded into SRplot. Finally, the overlapping genes of DETs and diAS were imported into Venn 2.1.0 and histograms were generated with the ggplot2 R package (v3.3.6).

### 2.7 qPCR and RT-PCR

T47D cells RNA in each group was extracted following the protocol of Trizol reagent kit (Cat#15596018CN, Invitrogen, Carlsbad, CA, United States), The RNA content was assessed using an ultra-microspectrophotomete. Then, complementary DNA (cDNA) was obtained from the RNA through the process of reverse transcription, utilizing a HiScript II Q RT SuperMix (Cat#R222-01; Epizyme, shanghai, China). Afterwards, SYBR-Green method was used for real-time quantitative cDNA amplification (Cat#Q711-02; Epizyme, shanghai, China). Finally, the relative mRNA levels have been determined to use the 2^−ΔΔCT^ method and standardized against GAPDH. RT-PCR primers that are intended to amplify two or more isoforms of various sizes are illustrated. The primers sequences of the genes for qPCR/RT-PCR are shown in Table 1. The ImageJ software was used to quantify the PCR results.

**TABLE 1:** Primers sequences of the genes used for qPCR and RT-PCR analysis.

Gene		Primer sequence (5′–3′)
*Tead2-202*	Forward	CAA​GGG​AAA​TCC​AGT​CCA​AGT​T
	Reverse	GGC​CTG​GAT​AGG​ACA​CAA​AGA​A
*Cenpx-202*	Forward	CAC​CTG​CAC​TTC​AAG​GAT​GAC​A
	Reverse	AGA​AAC​GCG​AGA​GGT​GGG​A
*Ncapd3-203*	Forward	GCC​AGA​CTT​TCC​CTG​ACA​TGT​T
	Reverse	GTC​AGC​AAT​TCC​TAC​GGC​AA
*Ncaph-201*	Forward	TCT​GTC​ACT​CGA​AGA​GCT​GTT​TCT
	Reverse	GAG​GCT​CAG​CTA​CCA​AGT​TTG​AC
*Paqr4-201*	Forward	AGG​TTC​CGA​GGC​TCA​AAG​G
	Reverse	AGC​TGG​TTT​CAT​CCG​GCA​CT
*Ncapd3*	Forward	GCA​GAG​ACA​CCA​GCA​GAG​GAG
	Reverse	CCA​ACA​GGT​CTT​CGT​CCA​TAT​TCC
*Gapdh*	Forward	GGA​AGC​TTG​TCA​TCA​ATG​GAA​ATC
	Reverse	TGA​TGA​CCC​TTT​TGG​CTC​CC

### 2.8 Western blot analysis

The Western blot analysis was carried out in accordance with the previously reported techniques (Wang P et al., 2024; Wang PY et al., 2024). In brief, protein samples ranging from 20 to 40 μg were analysed using a 10%–12% (w/v) SDS-PAGE gel (Cat#PG212/PG213; Epizyme, shanghai, China). The proteins that had been isolated were subjected to electroblotting and subsequently deposited onto a polyvinylidene difluoride (PVDF) membrane. The membrane was blocked using TBST solution that consisted of 5% nonfat milk or bovine serum albumin. Subsequently, the membranes were subjected to an overnight at 4°C with primary antibodies (xCT (Cat#26864-1-AP), GPX4 (Cat#30388-1-AP), HO-1 (Cat#10701-1-AP), NCAPD3 (Cat#16828-1-AP), 1:1000, ProteinTech Group, Chicago, United States). And its was subjected to incubation with secondary antibodies (Cat#L3032/L3012, 1:10,000, Signalway Antibody, Greenbelt, MD, United States) at ambient temperature. The visualisation of protein blots was achieved by the utilisation of an ECL system and the Image Lab detection system, manufactured by BioRad in Hercules, CA. GAPDH, β-actin, or α-tubulin (Cat#WL01114/WL01372/WL02296, 1:1000, Wanlei Biological Technology Co., Ltd., Shanghai, China) were employed to normalize the protein bands and examine them using ImageJ.

### 2.9 Statistics analysis

Statistical analysis was conducted using the GraphPad Prism (version 9.00). The experiments were repeated at least three times, and the data are expressed as the mean ± standard error of the mean (SEM). Statistical differences between two groups were analyzed using Student’s t-test, One-way ANOVA and Two-way ANOVA were used for comparison among multiple groups. *P < 0.05* was considered to indicate statistical significance.

## 3 Results

### 3.1 The analysis of differential alternative splicing (diAS) in THSG-treated T47D cells

**FIGURE 1:**
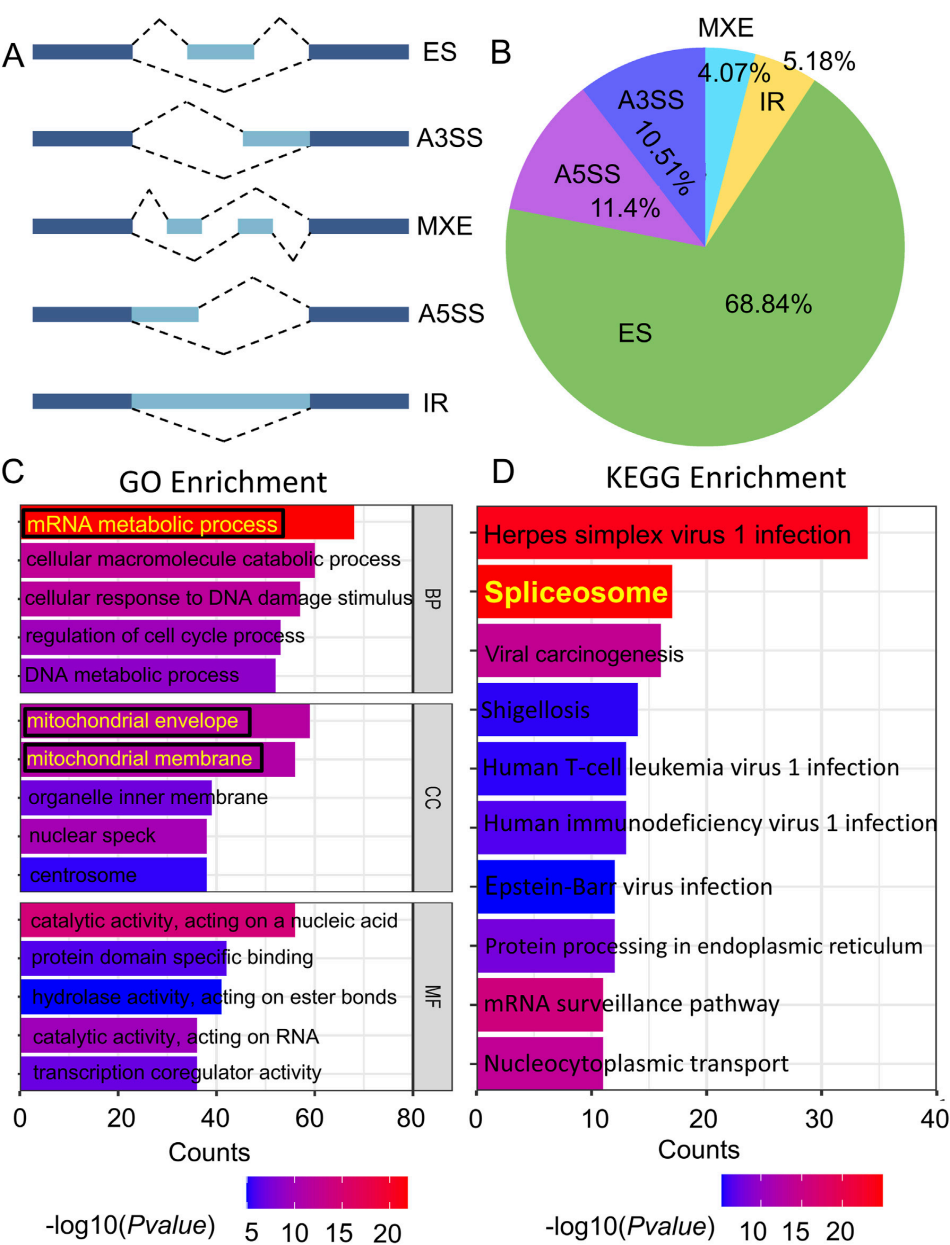
The analysis of differential alternative splicing (diAS) in T47D cells. **(A)** 5 types of Alternative splicing (AS). Exons are denoted by a dark blue color, while introns are denoted by pale blue. **(B)** The proportion of the five alternative splicing types in T47DT group. **(C)** Gene Ontology (GO) enrichment of the differential alternative splicing (diAS). **(D)** KEGG enrichment of diAS. The colour of the bar indicates the *P value* enriched into the pathway, while the length of the bar reflects the amount of genes.

Alternative splicing is an important factor in the development of protein variety. The different types of alternative splicing include exon skipping (ES), alternative 3′splice site (A3SS), mutually exclusive exon (MEX), alternative 5′splice site (A5SS), and intron retention (IR) (Figure 1A). As illustrated in Figure 1B, the classification of alternative splicing events in T47D cells following THSG treatment (T47DT) was shown. We observed that over half alternative splicing events were ES events. Furthermore, GO and KEGG enrichment analysis were performed to explore the differential alternative splicing (diAS). As shown in Figure 1C, it shown that the BP was mainly concentrated on the mRNA metabolic process and CC was mainly located in the mitochondrial envelope and mitochondrial membrane. Meanwhile, the *Spliceosome* was the most valuable pathway among the top 10 KEGG pathways (Figure 1D). Taken together, it indicated that THSG may inhibit T47D cells proliferation by regulating mRNA metabolic process and spliceosome. Nevertheless, the specific transcripts by which THSG exerts its effects have not been identified.

### 3.2 The overlapping transcripts analysis between DETs and diAS

**FIGURE 2:**
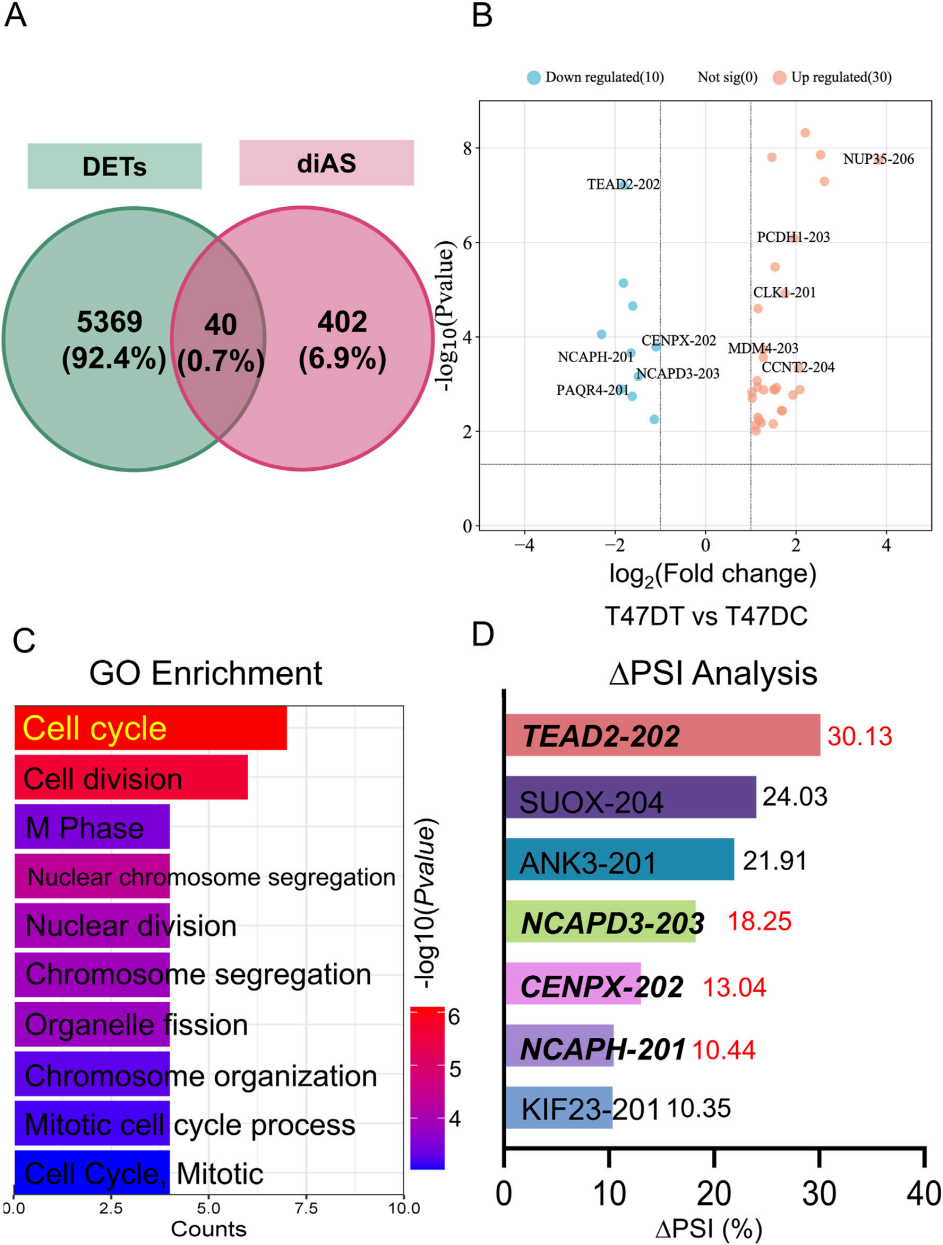
The overlapped transcripts analysis between DETs and diAS. **(A)** Venn programs of DETs and diAS. The green circle represented the DETs, and the red circle represented the diAS. **(B)** Volcano plot of overlapped genes in T47DT and T47DC. The blue (red) dots represented the downregulated (upregulated) genes in T47DT group. **(C)** GO enrichment analysis of overlapped transcripts. **(D)** ∆PSI analysis of downregulated transcripts in T47DT group. Abbreviations: GO, Gene Ontology; diAS, differential alternative splicing; AS, Alternative splicing; DETs, differential expression transcripts; PSI, Percent-spliced-in.

The overlapping transcripts (40) between the differentially-expressed transcripts (DETs) and differential alternative splicing events (diAS) related genes were shown in Figure 2A. Moreover, volcano plot was revealed in downregulated transcripts for *Tead2-202*, *Cenpx-202*, *Ncaph-201*, *Ncapd3-203*, and *Paqr4-201* or upregulated transcripts for *Unp35-206*, *Pcdh1-201*, *Clk-1-201*, *Mdm4-203*, and *Ccnt2-204* in THSG-treated groups (Figure 2B). Then, GO enrichment of overlapped transcripts (top 10) was performed in Figure 2C, and the top 3 pathways were cell cycle, cell division, and M phase. Meanwhile, the ∆PSI as the indicator ranked the downregulated transcripts (∆PSI > 0) as shown in Figure 2D.

**FIGURE 3:**
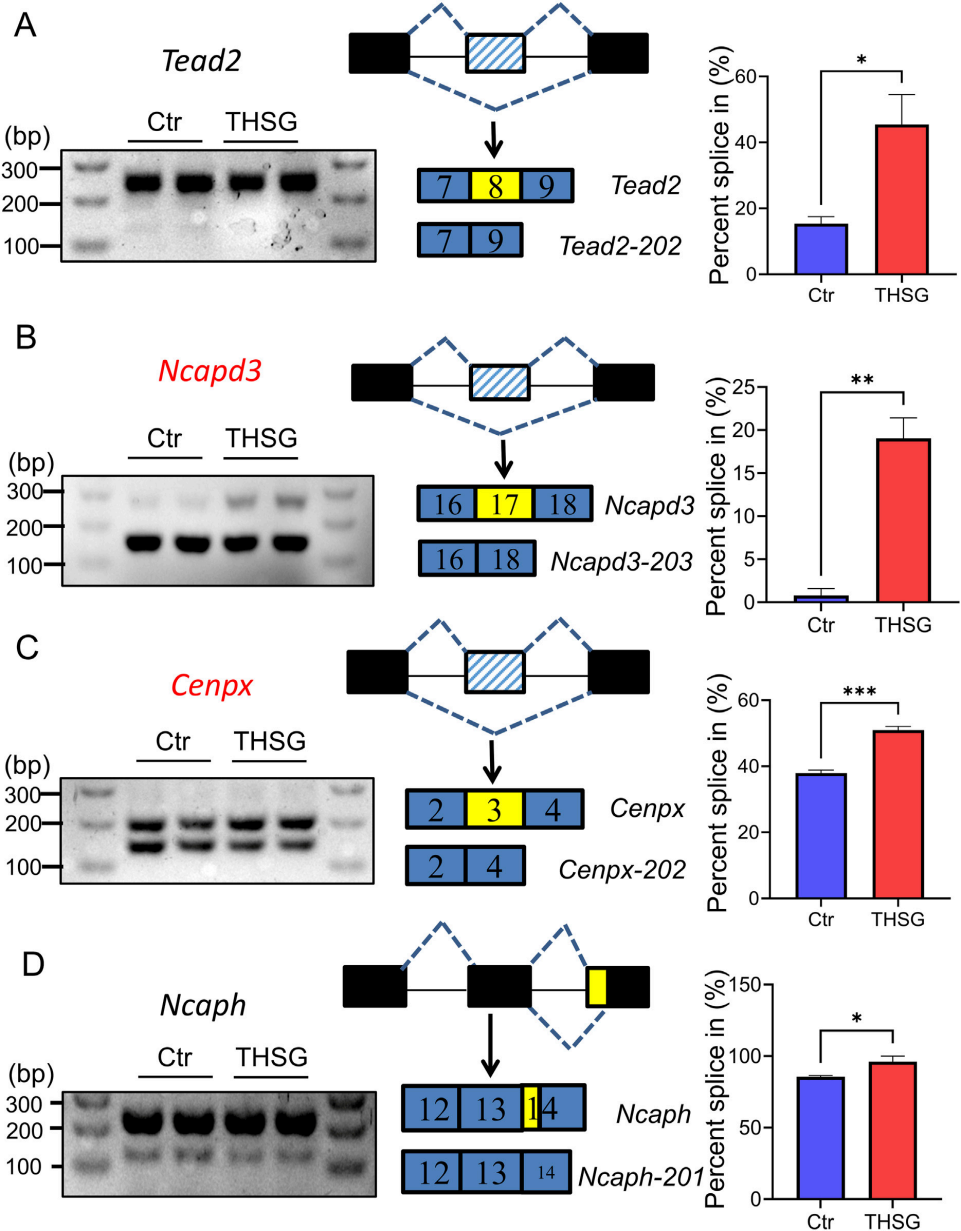
Type of alternative splicing was validated using RT-PCR. **(A)** *Tead2* and exon skipping (ES) **(B)** *Ncapd3* and exon skipping (ES) **(C)** *Cenpx* and exon skipping (ES) **(D)** *Ncaph* and exon skipping (ES). The black lines represent the regions of intron, the black (blue) rectangles represent the regions of exon, the yellow rectangles represent the regions of AS event. ^*^ *P < 0.05,* ^**^ *P < 0.01,* ^***^ *P < 0.001* vs the Ctr group.

**FIGURE 4:**
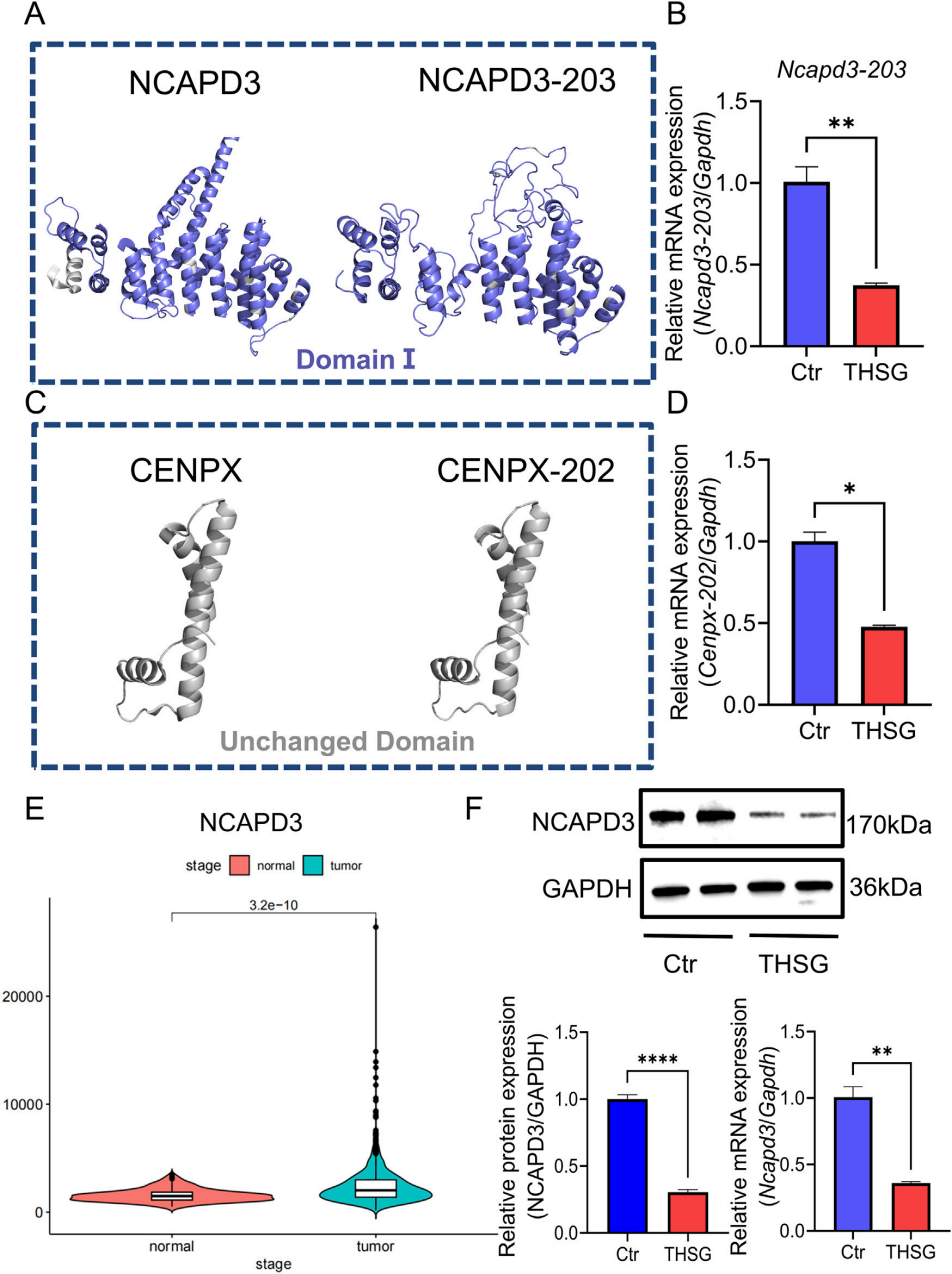
*Ncapd3* was a key gene for THSG to inhibit T47D cells proliferation. **(A, B)** Comparing the protein structures of NCAPD3 and NCAPD3-203, THSG treatment can inhibit the mRNA expression level of *Ncapd3-203* **(C, D)** Comparison of the protein structures of CENPX and CENPX-202. THSG treatment can inhibit the mRNA expression level of *Cenpx-202*. **(E)** NCAPD3 is highly expressed in breast cancer in TCGA database **(F)** The proteins (mRNA) expression levels were detected in Western blot and qPCR analysis, n = 3. ^*`^ *P < 0.05*, ^**^ *P < 0.01* vs the Ctr group.

Furthermore, Reverse Transcription-Polymerase Chain Reaction (RT-PCR) was performed to validate the AS changes of main transcripts (Figure 3). The inclusion reads of *Ncapd3* and *Cenpx2* were increased in THSG treatment, with the same as the results of RNA-seq data (Figures 3B, C). However, the inclusion reads of *Tead2* and *Ncaph* has no significant difference after THSG intervention (Figures 3A, D). Notably, the mRNA expression of *Ncapd3-203* and *Cenpx-201* were decreased in treatment of THSG (Figures 4B, D). Therefore, these results confirmed that the overlapping transcripts of DETs and diAS play an important roles in the cell cycle, and it was worthwhile to explore the transcripts of *Ncapd3* and *Cenpx* in further study.

### 3.3 *Ncapd3* was a key gene for THSG to inhibit T47D cells proliferation

Given the critical role of protein-level analyses in understanding biological processes, we predicted the protein structure of NCAPD3 (NCAPD3-203) and CENPX (CENPX-202). Compared with NCAPD3, the altered region of NCAPD3-203 is located within the domain (Figures 4A, B), whereas the change of CENPX-202 is located within the non-structural domain (Figures 4C, D). Therefore, it will indicate that the alternative splicing event of *Ncapd3* will be more valuable for research.

As shown in Figure 4E, *Ncapd3* is highly expressed in breast cancer, and the protein levels of NCAPD3 and the mRNA expression of *Ncapd3* were decreased in THSG group (Figure 4F). Collectively, the findings corroborated the elevated expression of NCAPD3 in T47D cells. And THSG could inhibit the protein (mRNA) expression level of NCAPD3 (*Ncapd3*).

### 3.4 THSG has the potential to trigger ferroptosis in T47D cells

**FIGURE 5:**
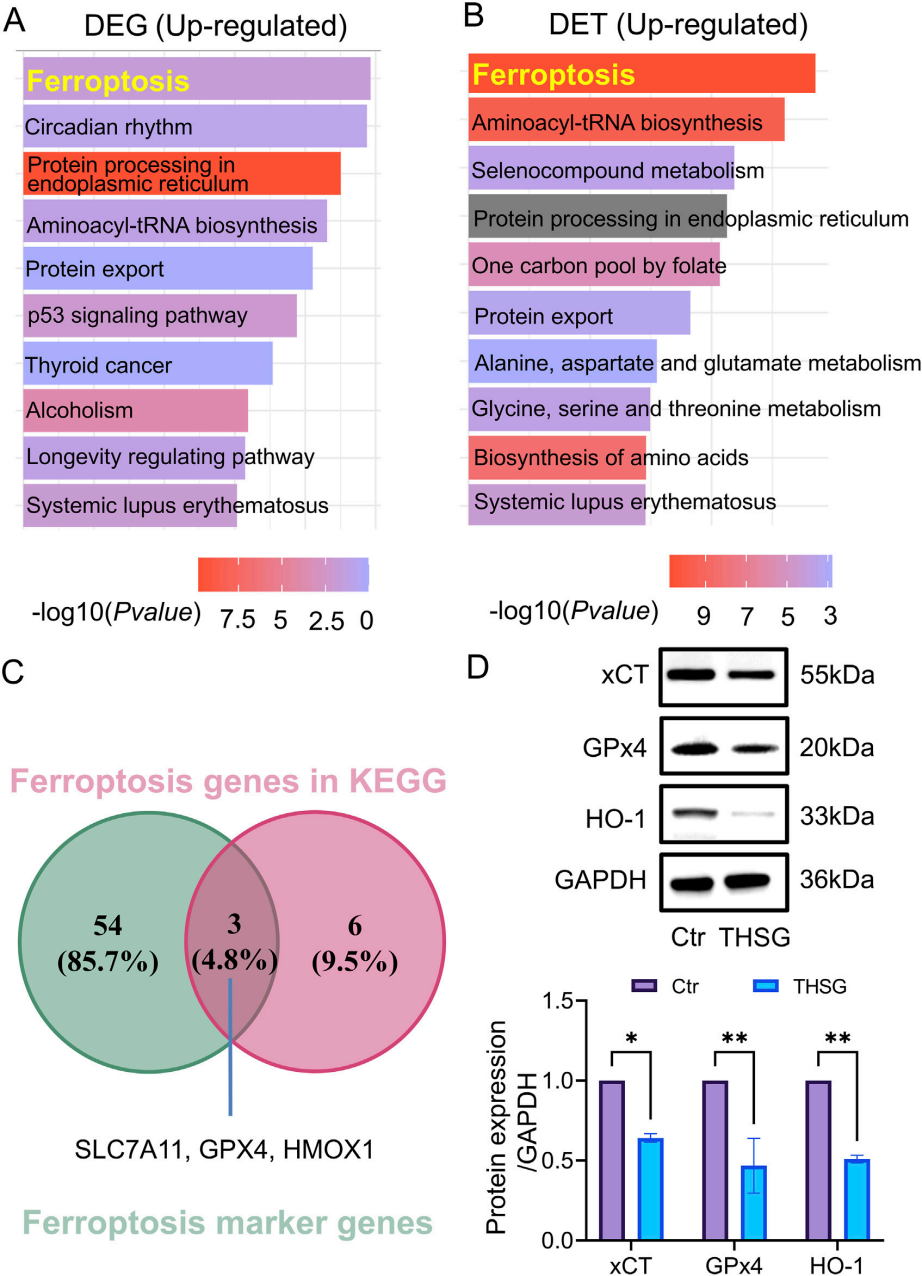
Ferroptosis was the hub pathway for THSG treating for T47D cells. **(A)** KEGG analysis was conducted in DEGs upregulated genes **(B)** KEGG analysis was conducted in DETs upregulated transcripts. **(C)** The overlapping genes (*Slc7a11, Gpx4, Hmox1*) were detected in ferroptosis genes in KEGG and ferroptosis marker genes. **(D)** The protein level of xCT, GPx4 and HO-1 was conducted using WB, *n = 3*. ^*^ *P < 0.05,* ^**^ *P < 0.01* vs the Ctr group.

To investigate the potential mechanism of THSG suppress T47D cells proliferation, differentially-expressed genes (DEGs) and differentially-expressed transcripts (DETs) were selected to analysis. Compared with the KEGG enrichment of upregulated genes (transcripts) in DEGs and DETs after THSG treatment of T47D cells (Figures 5A,B), ferroptosis was the common pathway and was in the first place ranked in rich factor. However, the *P value* of the ferroptosis pathway in the upregulated genes of DETs is smaller, which will indicate that the ferroptosis pathway is more likely to be enriched in DETs.

To further explore the specific genes in ferroptosis pathway, the ferroptosis marker genes were selected in FerrDb V1 (http://www.zhounan.org/ferrdb/legacy/) database. It was worth noting that 3 overlapping genes (*Slc7a11*, *Gpx4*, *Hmox1*) were detected in ferroptosis genes in KEGG and ferroptosis marker genes (Figure 5C). In addition, the protein level of xCT, GPx4, and HO-1 was decreased in THSG treatment (Figure 5D). Altogether, it was determined that THSG treatment triggers ferroptosis in T47D cells.

### 3.5 THSG could induce cell cycle arrest in T47D cells

**FIGURE 6:**
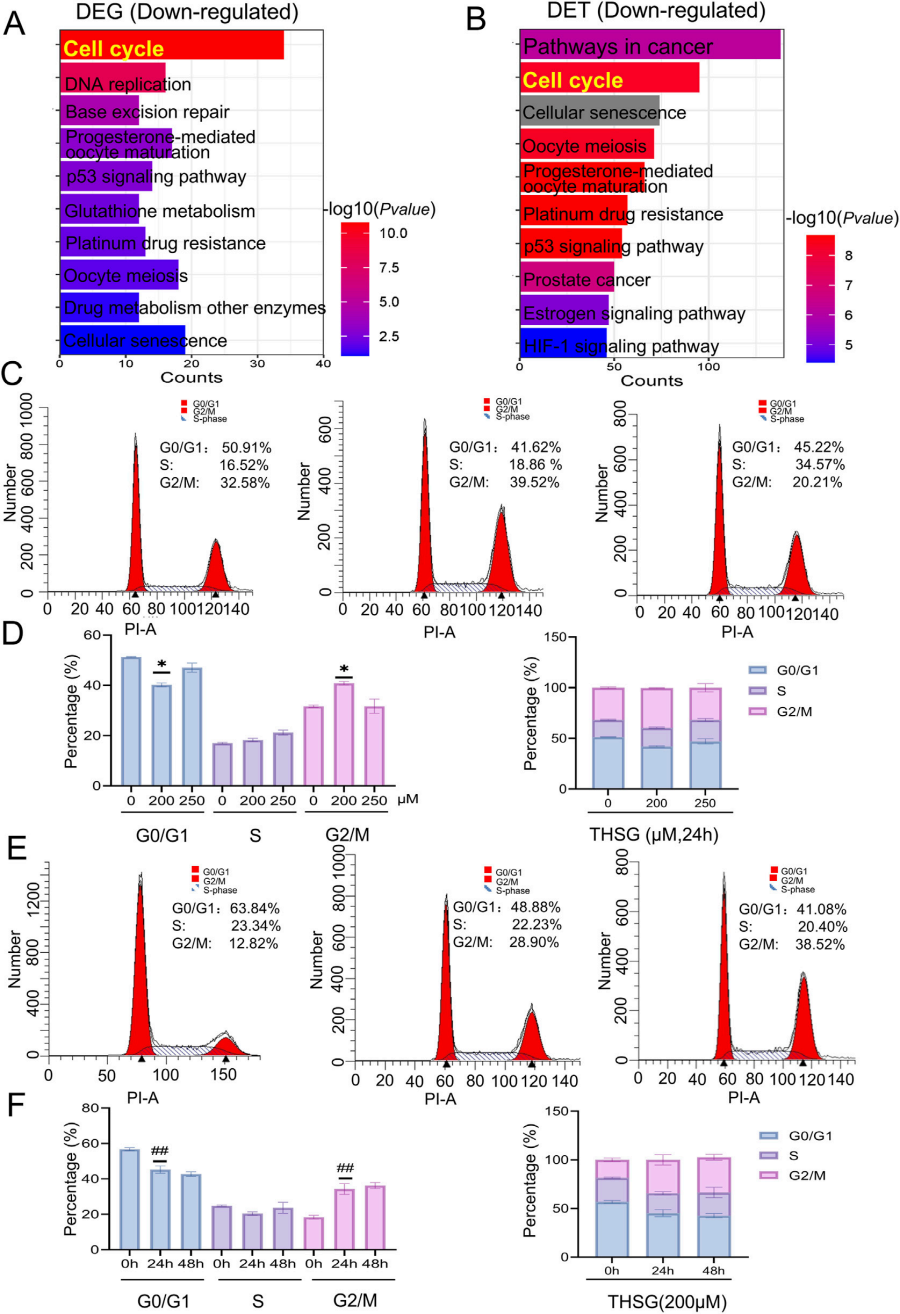
THSG could provoke an arrest of cell cycle in T47D cells. **(A)** KEGG analysis of DEGs downregulated genes **(B)** KEGG analysis of DETs downregulated genes.**(C)** Cell cycle diagram after treatment with THSG (200 and 250 μmol/L) at 24 h by flow cytometry. **(D)** Cell cycle phase distribution (%) treated by 200 and 250 μmol/L THSG at 24 h, n = 3. ^*^ *P < 0.05,* vs the 0 μM group. **(E)** Cell cycle diagram after THSG-treated (200 μmol/L) at 24h and 48 h by flow cytometry. **(F)** Cell cycle stage distribution (%) after treated with 200 μmol/L THSG at 24 and 48 h, n = 3. ^*##*^ *P < 0.01,* vs the 0 h.

Given that abnormalities in the cell cycle are a principal cause of cancer development and progression. Consistent with the reports, cell cycle played an vital roles in enriched for downregulated genes of DEGs and DETs (Figures 6A, B). Further, we conducted flow cytometry analysis to investigate the specific mechanisms about cell cycle in T47D cell death. As shown in Figures 6C–F, cell cycle arrest was notably induced by THSG treatment at various doses and time points, especially in the G2/M phase. As the concentration of THSG increased for 24h, the percentage of cells in the G2/M phase was increased significantly, whereas the percentage of cells in the S and G0/G1 phases was decreased (Figures 6C, D). Furthermore, time is a crucial determinant of the cell cycle. With time, there was also a significant increase in the percentage of cells in the G2/M stage in 24h and 48 h (Figures 6E, F). Notably, when 200 μM THSG was used to treat T47D cells for 24 h, THSG had the most significant effect on the cell cycle arrest.

Collectively, the KEGG enrichment results of DEGs and DETs preliminarily showed that THSG could promote the occurrence of cell cycle arrest in T47D cells.

### 3.6 Silence of NCAPD3 accelerates THSG-induced ferroptosis in T47D cells

**FIGURE 7:**
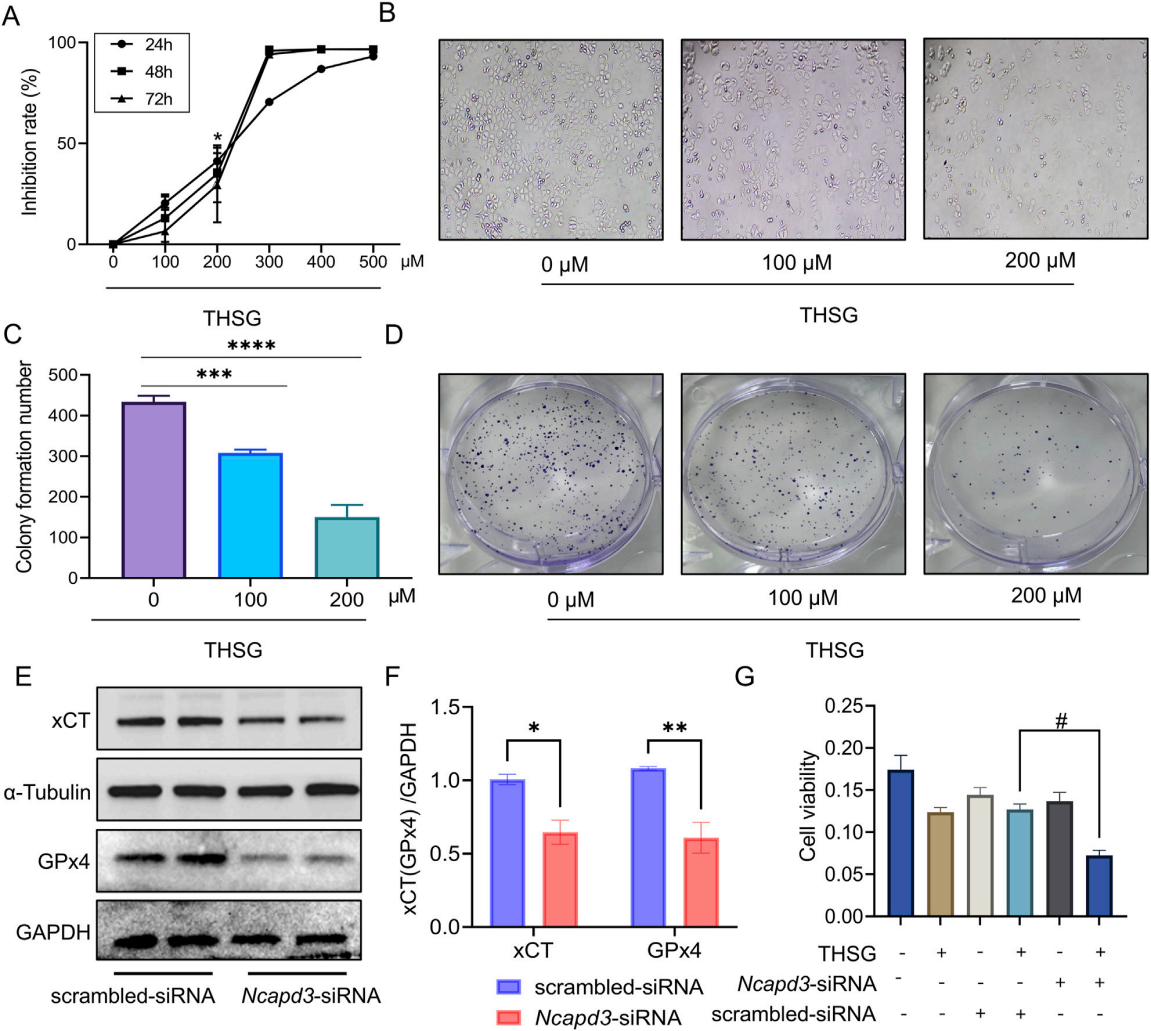
Reduction of NCAPD3 accelerates THSG-induced ferroptosis in T47D cells. **(A, B)** MTT experiment reveals the dose-time relationship of THSG in inhibiting T47D cells proliferation, n = 3. **(C, D)** Clone formation experiment. ^*^ *P < 0.05*, ^***^ *P < 0.001*, ^****^ *P < 0.0001* vs the 0 μM group. **(E, F)** The protein expression of xCT and GPx4 was shown in scrambled-siRNA and NCAPD3-siRNA group, *n = 4*. **(G)** T47D cells viability was detected using MTT assay, *n = 3*. ^*^ *P < 0.05,* ^**^ *P < 0.01*, vs the scrambled-siRNA group. ^*#*^ *P < 0.05* vs the THSG + scrambled-siRNA group.

To explore the effects of THSG on T47D cells, we observed a dose-time independent increasing in the inhibitory rate of THSG. It is worth noting that the T47D cells is significantly inhibited when 200 μmol/L THSG is used (Figures 7A, B). Moreover, the clone formation experiment showed that 200 μmol/L THSG treatment has a significant inhibitory effect on the proliferation of T47D cells, and this concentration was used in subsequent experiments (Figures 7C, D).

We further investigated the ferroptosis-related proteins to determine the role of NCAPD3 in ferroptosis in T47D cells. Notably, the expression levels of xCT and GPx4 proteins were reduced after knockdown of NCAPD3 (Figures 7E, F). When THSG was administered, the survival rate of T47D cells was decreased significantly (Figure 7G). Therefore, it would indicated that THSG could accelerate ferroptosis of T47D cells and thereby inhibited cell proliferation.

## 4 Discussion

To determine the mechanism by which THSG inhibits T47D cells proliferation, we used full-length transcriptome sequencing to investigate the differences in gene expression. In particular, *Ncapd3* was the hub gene that THSG treated for T47D cells proliferation. Moreover, domain analysis and experimental verification indicated that The expression of the protein may be inhibited by THSG binding to the NCAPD3 domains. Furthermore, the ferroptosis and cell cycle were the highlight pathways in DEGs and DETs analysis of KEGG enrichment, and then THSG triggered ferroptosis and induced G2/M cell cycle arrest in T47D cells. Further studies showed that *Ncapd3*-siRNA administration could decrease the expression levels of xCT and GPx4, and THSG was able to inhibit the proliferation and clone formation of T47D cells. In this study, THSG could trigger T47D cells ferroptosis by down-regulating the expression of *Ncapd3*, and it could induce cell cycle arrest in G2/M stage.

Alternative splicing is an important post-transcriptional regulatory mechanism that can regulate the translation of mRNA isoforms and induce protein diversity, thereby expanding gene coding capacity. Statistics indicated that the incidence and advancement of breast cancer are significantly associated with alternative splicing, which also serves as a viable therapeutic target (Yang Q et al., 2019). 5 types of alternative splicing were detected after THSG intervention, and exon skipping (ES) accounted for the highest proportion, which is consistent with the most studies. Subsequently, RNA sequencing data and experimental validation were employed to further investigate the intersection of differentially expressed transcripts and differential alternative splicing, and *Ncapd3-203* may be one of the key transcripts through which THSG inhibits T47D cells proliferation.

Non-SMC condensin II complex subunit D3 (NCAPD3) is one of the three non-SMC subunits of the condensin II complex and plays a crucial role in the condensation and segregation of mitotic chromosomes (Ono SJ et al., 2003). High expression of NCAPD3 has been reported to cause chromosomal instability in mouse models of colorectal cancer, thereby promoting cancer progression (Pussila M et al., 2018). While in pancreatic cancer, NCAPD3 serves as a predictive indicator for clinical trials (Dawkins JB et al., 2016). These results all indicate that NCAPD3 may play a role in the process of cancer promotion. Nevertheless, there is no knowledge of NCAPD3 contribution to the development of breast cancer. It is worth noting that NCAPD3 was highly expressed in breast cancer, and THSG inhibited the expression level of NCAPD3 in T47D cells. Further studies have shown that when siNCAPD3 is given, THSG can inhibit the xCT and GPx4 protein expression level, thereby promoting ferroptosis of T47D cells.

Current study evidence indicates that the triggering to ferroptosis has the potential to be an approach employable for cancer therapy, particularly in eradicating aggressive malignancies resistant to conventional treatments (Liang C et al., 2019; Li FJ et al., 2024). System Xc⁻ (xCT) was first identified in human fetal fibroblasts (Bannai and Kitamura, 1980), the expression of xCT is regulated by multiple factors at multiple levels, including transcription, post-transcription and translation. Two components make up System Xc-. One is the heavy chain, and the other is the light chain (xCT). These subunits are linked by an extracellular covalent disulfide bond. The transport function of system xc-requires the combined participation of both the heavy and light chain subunits (Lim and Donaldson, 2011). Studies have shown that the expression levels of the two subunits of System Xc⁻ determine GPx4 expression in breast cancer (BC) cells (Lee N et al., 2021). Therefore, targeting SystemXc⁻/GPX4 to induce ferroptosis in breast cancer cells is a possible therapeutic strategy. Our research has found that 2,3,5,4’-Tetrahydroxystilbene glucoside (THSG) downregulated the expression of xCT and GPx4, promoting ferroptosis in T47D cells. Further analysis revealed that THSG also arrests T47D cells at the G2/M phase.

Recent studies indicate that traditional Chinese medicine (TCM) plays a significant role in regulating ferroptosis, with multiple natural compounds derived from TCM shown to induce ferroptosis (Lou Y et al., 2022; Wu et al., 2022). For example, Lycium barbarum polysaccharide (LBP), an extract from the Chinese herbal fruit Lycium barbarum, induces ferroptosis in breast cancer cells by modulating the xCT/GPX4 pathway and reducing glutathione (GSH) synthesis (DU X et al., 2022). Similarly, glycyrrhetinic acid (GA) exacerbates ferroptosis in breast cancer by inhibiting xCT expression and GPX4 activity, depleting GSH (Wen Y et al., 2021). Notably, our results demonstrate that THSG reduces xCT and GPX4 protein levels while increasing GSH content in T47D cells, potentially due to its strong antioxidant properties (Xiang et al., 2014; Lin HY et al., 2022). Additionally, the effects of THSG on GSH in cancer have not been previously reported in the literature. Therefore, we propose that THSG may prevent the breast cancer growth by decreasing xCT and GPX4 expression and arresting breast cancer cells in the G2/M phase.

In addition, it is worth affirming that the single use of diphenylethylene glycosides does not produce obvious toxic effects on cells or experimental animals (Yu J et al., 2011; Shen J et al., 2018; Zhang SH et al., 2009), but some studies have shown that when diphenylethylene glycosides are used in combination with other toxic drugs, they affect related metabolic enzymes and enhance the toxic side effects of other drugs (Ma J et al., 2013). This suggests that there may be interactions between different components, which affect related drug-metabolizing enzymes, leading to toxic side effects. Therefore, special attention should be paid to the combined use of diphenylethylene glycosides with other drugs in clinical practice. In addition, comprehensive and systematic toxicological studies are needed to understand the toxicity and mechanism of action of diphenylethylene glycosides.

**FIGURE 8:**
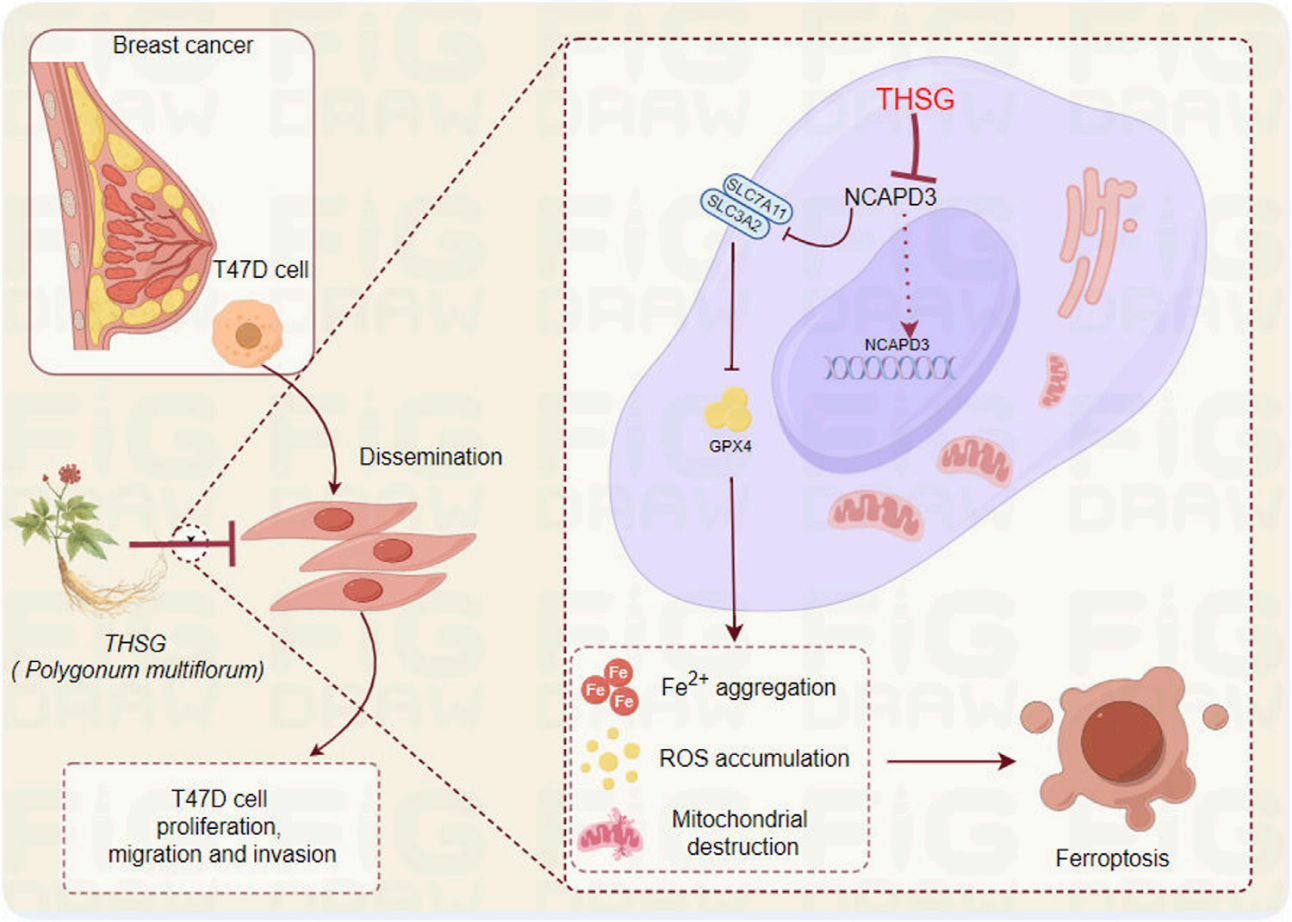
The hypothesis of by which THSG inhibiting T47D cell proliferation (The Figure was performed by Figdraw (https://www.figdraw.com/#/).

In conclusion, THSG could inhibit the expression of ferroptosis-related proteins (xCT and GPx4) by inhibiting the expression of NCAPD3, thereby inhibiting the proliferation of T47D cells, which may be related to the arrest of the cell cycle (G2/M phase) (Figure 8). However, more breast cancer-related cell lines and *in vivo* animal experiments should be further explored to verify the potential clinical application of THSG.

## Data Availability

The original contributions presented in the study are publicly available. This data can be found here: 10.6084/m9.figshare.28262996.
